# Familiarity with, perceptions of and attitudes toward butterflies of urban park users in megacities across East and Southeast Asia

**DOI:** 10.1098/rsos.220161

**Published:** 2022-11-09

**Authors:** Voon-Ching Lim, Kong-Wah Sing, Kwek Yan Chong, Narong Jaturas, Hui Dong, Ping-Shin Lee, Nguyen Thien Tao, Dzung Trung Le, Timothy C. Bonebrake, Toby P. N. Tsang, Leo Chu, Guo-Jie Brandon-Mong, Wye-Lup Kong, Masashi Soga, John-James Wilson

**Affiliations:** ^1^ School of Science, Monash University Malaysia, 47500 Bandar Sunway, Selangor, Malaysia; ^2^ South China DNA Barcoding Center, Kunming Institute of Zoology, Chinese Academy of Sciences, Kunming, Yunnan 650223, People's Republic of China; ^3^ State Key Laboratory of Genetic Resources and Evolution, Kunming Institute of Zoology, Chinese Academy of Sciences, Kunming, Yunnan 650223, People's Republic of China; ^4^ Department of Biological Sciences, National University of Singapore, 16 Science Drive 4, Singapore 117558, Republic of Singapore; ^5^ Singapore Botanic Gardens, National Parks Board, 1 Cluny Road, Singapore 259569, Republic of Singapore; ^6^ Department of Microbiology and Parasitology, Faculty of Medical Science, Naresuan University, Phitsanulok, Thailand; ^7^ Fairy Lake Botanical Garden, Shenzhen & Chinese Academy of Sciences, Shenzhen, People's Republic of China; ^8^ Anhui Provincial Key Laboratory of the Conservation and Exploitation of Biological Resources, College of Life Sciences, Anhui Normal University, Wuhu, Anhui 24100, People's Republic of China; ^9^ Vietnam Academy of Science and Technology, Institute of Genome Research, 18 Hoang Quoc Viet Road, Cau Giay, Hanoi, Vietnam; ^10^ Vietnam Academy of Science and Technology, Graduate University of Science and Technology, 18 Hoang Quoc Viet, Cau Giay, Hanoi, Vietnam; ^11^ Ministry of Education and Training, 35 Dai Co Viet Road, Hai Ba Trung District, Vietnam; ^12^ School of Biological Sciences, The University of Hong Kong, Hong Kong; ^13^ Department of History and Philosophy of Science, University of Cambridge, Free School Lane, Cambridge CB2 3RH, UK; ^14^ Department of Life Science, National Taiwan Normal University, Taipei, Taiwan; ^15^ Biodiversity Program, Taiwan International Graduate Program, Academia Sinica and National Taiwan Normal University, Taipei, Taiwan; ^16^ Graduate School of Agricultural and Life Sciences, The University of Tokyo, Tokyo, Japan; ^17^ Vertebrate Zoology at World Museum, National Museums Liverpool, William Brown Street, Liverpool L3 8EN, UK; ^18^ Department of Biology, Faculty of Science, Chulalongkorn University, 10330 Bangkok, Thailand; ^19^ Department of Biological Sciences, University of Toronto-Scarborough, Toronto, Ontario, Canada; ^20^ National Primate Research Center of Thailand, Chulalongkorn University, 18110, Saraburi, Thailand

**Keywords:** butterflies, conservation, human–wildlife interactions, megacities, nature experiences, urban wildlife

## Abstract

Perceptions of, and attitudes toward, wildlife are influenced by exposure to, and direct experiences with, nature. Butterflies are a conspicuous and ubiquitous component of urban nature across megacities that are highly urbanized with little opportunity for human–nature interactions. We evaluated public familiarity with, perceptions of and attitudes toward butterflies across nine megacities in East and Southeast Asia through face-to-face interviews with 1774 urban park users. A total of 79% of respondents had seen butterflies in their cities mostly in urban parks, indicating widespread familiarity with butterflies. Those who had seen butterflies also had higher perceptions of butterflies, whereas greater than 50% of respondents had positive attitudes toward butterflies. Frequent visits to natural places in urban neighbourhoods was associated with (i) sightings of caterpillars, indicating increased familiarity with urban wildlife, and (ii) increased connectedness to nature. We found two significant positive relationships: (i) between connectedness to nature and attitudes toward butterflies and (ii) between connectedness to nature and perceptions of butterflies, firmly linking parks users' thoughts and feelings about butterflies with their view of nature. This suggests that butterflies in urban parks can play a key role in building connectedness to nature and consequently pro-environmental behaviours and support for wildlife conservation among urban residents.

## 1. Introduction

Earth is now an ‘urban planet’ [1] and dramatic shifts in land use continue unabated. This is especially true in East and Southeast Asia where cities are transforming into ‘megacities’ [2]. Megacities are ‘a cluster of highly networked urban settlements anchored by one or more large cities' which have experienced rapid urbanization and human population growth [3]. Natural habitats are replaced by impermeable structures, and natural resources from peri-urban areas are exploited to support economic activities in cities, resulting in biotic homogenization on a massive scale [4]. Although urbanization largely impacts biodiversity adversely, some man-made habitats such as urban parks may function as surrogates for habitats otherwise absent from intensively managed land, hence supporting wildlife populations [5] and providing essential ecosystem services [6].

While it is important to examine how urban green spaces support biodiversity and ecological processes, understanding human perceptions and use of ‘urban nature’ is equally crucial for developing conservation strategies that (i) benefit biodiversity and well-being of urban residents [7] and (ii) are likely to be supported by residents [8]. Consequently, understanding urban residents' perceptions of, and attitudes toward, urban nature is a rapidly developing area of research [9–12]. The ‘pigeon paradox’ by Dunn *et al.* [8] posits that the frequency and quality of interactions with wildlife (including non-native species) in cities impact residents' pro-environmental action and will ultimately determine the success of efforts to address the planetary biodiversity emergency [13,14]. The interactions happening in urban green spaces such as parks will shape residents’ emotional connection with nature, how they respond toward wildlife, and consequently their sensitivity to broader environmental issues [12–16]. For example, undergraduates in Tokyo who positively valued the natural environment did not find birds and butterflies in their neighbourhoods troublesome but tended to appreciate these urban species for their roles in mind relaxation [12]. In Singapore, residents who were more connected to nature tended to disagree that birds were visually unpleasant, aggressive and damage personal property [17].

Perceptions of, and attitudes toward, wildlife are influenced by exposure to, or direct experiences with, ‘nature’ [8,12,14,18–20]. Urban wildlife is often regarded as ‘good’ or ‘bad’, and positive, neutral, and negative sociocultural attitudes toward wildlife have persisted through history across the Asia-Pacific region [21–23]. For example, birds are generally regarded positively for their ecosystem services, but negative regard of birds due to their ecosystem disservices (e.g. faeces and noises) can result in harmful actions toward birds [17,24]. Nevertheless, improved knowledge and perceptions of urban wildlife can foster positive attitudes toward them [25].

Butterflies are colourful, conspicuous and ubiquitous in every megacity in East and Southeast Asia (e.g. [26–30]). While a growing number of citizen science projects in this region use butterflies as flagship species to promote biodiversity conservation in urban areas (e.g. [19,31,32]), human perceptions of butterflies and the extent this influences public support for biodiversity conservation across the region remain unexplored. In this study, we evaluated public familiarity with, perceptions of, and attitudes toward butterflies in nine megacities across East and Southeast Asia through a face-to-face questionnaire with urban park users.

## 2. Material and methods

### 2.1. Questionnaire design

**Figure 1.**
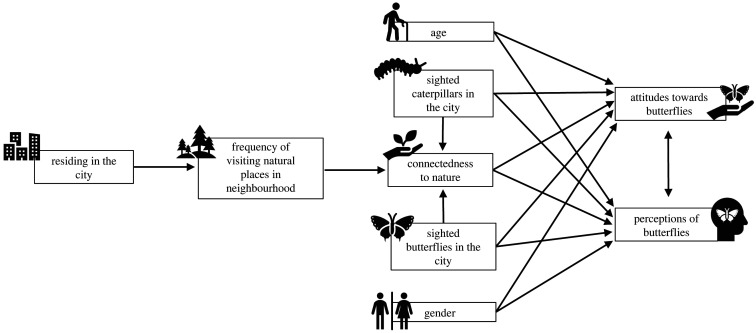
Conceptual framework of hypothetical relationships between potential explanatory variables (at the start of arrows) and response variables (at the end of arrows). Here we hypothesized that (1) city residents tend to visit parks near their homes; (2) visiting natural places (i.e. parks) in neighbourhood and having seen butterflies and/or caterpillars foster connectedness to nature; (3) park users with higher connectedness to nature have more positive perceptions of, and attitudes toward, butterflies; (4) gender, age and sightings of butterflies and/or caterpillars influence perceptions of, and attitudes toward, butterflies; (5) perceptions of butterflies and attitudes toward butterflies may be correlated.

With reference to previous literature [12,14,24,33–35], we conceptualized hypothetical relationships between potential explanatory and response variables (figure 1). Using our conceptualized hypothetical model and questionnaires used in previous studies [12,33,36], we developed a questionnaire (electronic supplementary material, S1) to evaluate urban park users' familiarity with, perceptions of, and attitudes toward butterflies, and the park users’ connectedness to nature. The questionnaire was first written in English, then translated to Malay, simplified Chinese, Thai, and Vietnamese. In no version of the questionnaire did we elaborate on differences between butterflies and moths or explain that caterpillars are lepidopteran larvae. A pilot study using a partial version of the final questionnaire was conducted in Kuala Lumpur, Malaysia in April 2016 to assess the comprehensibility and suitability of the statements (results were excluded from the present analyses).

The final questionnaire comprised six parts. Part A contained five questions to elicit the socio-demographic background of the respondents. Part B contained six questions to understand the respondents' familiarity with butterflies. Parts C, D and E each consisted of six statements, each requiring respondents to choose ‘strongly agree’, ‘agree’, ‘no opinion/not sure’, ‘disagree’ or ‘strongly disagree’ as their response. Part C aimed to examine respondents' perceptions of butterflies (i.e. an understanding of butterflies as an integral part of the city). Part D.i examined the respondents' attitudes toward butterflies (i.e. a measure of their liking of butterflies), and D.ii included two open-ended questions to obtain respondents' preferred and disliked qualitative characteristics of butterflies. Part E explored the respondents' connectedness to nature.

### 2.2. Questionnaire implementation

**Figure 2.**
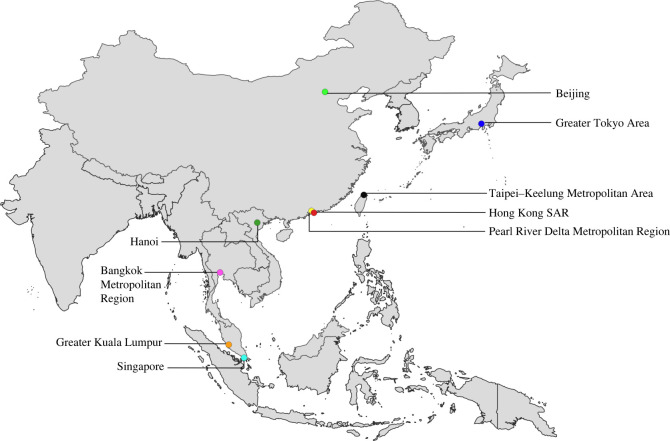
Locations of megacities across East and Southeast Asia where questionnaires were implemented. The map was created using shapefiles publicly available at https://www.cdc.gov/epiinfo/support/downloads/shapefiles.html and edited using QGIS software (https://qgis.org/en/site/).

We deployed our questionnaire at urban parks in nine megacities (used interchangeably with ‘city’ below) across East and Southeast Asia (figure 2) between 2017 and 2018. Parks that were open to the public and that receive a large number of visitors were selected. When in the parks, we approached potential respondents, introduced ourselves as researchers conducting a questionnaire, and asked if they were willing to take part. If they declined, we moved on to another potential respondent. If they consented, we briefed them that the purpose of the research was ‘to examine human interactions with butterflies in natural places in their neighbourhood’. Respondents were informed that their responses would remain anonymous, there was no risk nor harm associated with the questionnaire, and they were free to withdraw from answering at any point. In most cases, we asked the respondents the questions verbally and wrote down their answers ourselves. Occasionally when there were groups of several respondents, some would read the questions and write down their answers themselves, while remaining in our vicinity. For certain questions and statements that respondents did not wish to answer, the responses were recorded as ‘N/A’, denoting ‘not answered’.

### 2.3. Data analyses

Of 1774 completed questionnaires from nine megacities, 218 were excluded from further analyses due to: (i) responses with unanswered questions, (ii) >5 N/A responses, (iii) illegible or inconsistent responses and (iv) responses from individuals aged less than 18 years old. 1556 completed questionnaires were retained for subsequent analyses. For Parts C, D.i and E, the responses were transcribed as a 5-point Likert scale (‘strongly agree’ = 5, ‘agree’ = 4, ‘not sure’ = 3, ‘disagree’ = 2 and ‘strongly disagree’ = 1) with negative statements being reversely scored.

Analyses were performed in R v. 4.0.2 [37]. For Part D.ii, qualitative responses indicating preference and distaste for butterflies were visualized as word clouds using the ‘wordcloud’ package [38]. Pearson's correlation coefficient (less than 0.5) indicated no correlation between Parts C, D.i and E, against Part A. Using the function ‘cronbach.alpha()’ in the package ‘ltm’ [39], the Cronbach's alpha values calculated for Parts C and E were 0.816 and 0.884, indicating high internal consistency among the statements [40]. Therefore, the scores for all statements in Parts C and E were averaged to provide a single mean score for C = ‘perception of butterflies', and E = ‘connectedness to nature’ following Soga *et al*. [12]. For D.i, the Cronbach's alpha value was initially 0.611 but improved to 0.829 after the removal of statement D3: ‘I think butterflies are dangerous because their caterpillars can be poisonous'. Consequently, responses for D3 were excluded when averaging the scores for all other statements in Part D to provide a single mean score for D = ‘attitude toward butterflies’. For Part B, the calculated Cronbach's alpha value was consistently below 0.5 even after the removal of several statements. Therefore, only statements B1: ‘have you seen butterflies in this city?’ and B5: ‘have you seen caterpillars in this city?’ with binary responses (no = 0, yes = 1) were used in subsequent statistical analyses. Consequently, a total of 1309 completed questionnaires were used for linear mixed effects modelling (LMM) and piecewise structural equation modelling (SEM), with the hypothetical relationships (figure 1) as basic models.

LMM was performed to identify explanatory variables influencing response variables using the ‘lme4’ package [41] (figure 1). The random factor for the models was the location of the surveys (i.e. the megacity which was denoted as ‘City’ in the models). A global model (where all explanatory variables were present) was first built to identify the explanatory variables that influence the response variables using function ‘lmer()’ with maximum-likelihood method (REML = FALSE). The function ‘dredge()’ in the MuMIn package [42] was then used to conduct multi-model inference from all possible combinations of explanatory variables for each response variable and models with the lowest Akaike information criterion (AIC), lowest delta AIC and highest AIC weight were chosen as the best set of models for identifying the important explanatory variables. Delta AIC lower than 2 indicates substantial evidence to support the candidate model whereas the AIC weight indicates the probability that the candidate model is the best among all the models considered [43]. For *post hoc* analyses, independent *t-*tests were conducted for explanatory variables with two samples (i.e. male and female). Kruskal–Wallis tests and multiple comparison using Dunn's test with Benjamini–Hochberg adjustment for two-sided test (note that a two-sided *p*-value less than 0.05 was considered significant in this case) were conducted for explanatory variables with more than two samples (i.e. age groups).

SEM was conducted to further examine the relationships between the explanatory and response variables using the ‘piecewiseSEM’ package [44]. Based on the variables present in the best set of models resulting from the LMM analyses, we constructed an SEM model with LMM as the choice of regression using function ‘psem(lmer())’. The variables were converted to numeric binary (e.g. 0 = female and 1 = male) or ordinal (1 = strongly disagree, 2 = disagree, 3 = not sure, 4 = agree and 5 = strongly agree) categories following Lefcheck [45]. Based on the hypothetical relationships (figure 1) and LMM results, ‘perception of butterflies' and ‘attitude toward butterflies’ were considered to be correlated rather than directly causally associated and were modelled with correlated errors. The model was checked for the following criteria: (i) the variables were conditionally independent based on tests of directed separation (*p*-value > 0.05) and (ii) the structure of the SEM model is supported by the data without potential missing paths based on Fisher's C test (*p*-value > 0.05) [45].

The final SEM model was assessed with the comparative fit index (CFI), Tucker–Lewis index (TLI), root mean square error of approximation (RMSEA) and standardized root mean square residual (SRMR) using the function ‘fitMeasures()’ in the lavaan package [46]. CFI measures the relative improvement in fit from the baseline model to the postulated model, TLI measures a relative reduction in misfit per degree of freedom, while RMSEA measures the discrepancy due to the approximation per degree of freedom [47]. SRMR quantifies the discrepancies between the sample covariances and the implied ones derived from the parameters [48].

## 3. Results

**Figure 3.**
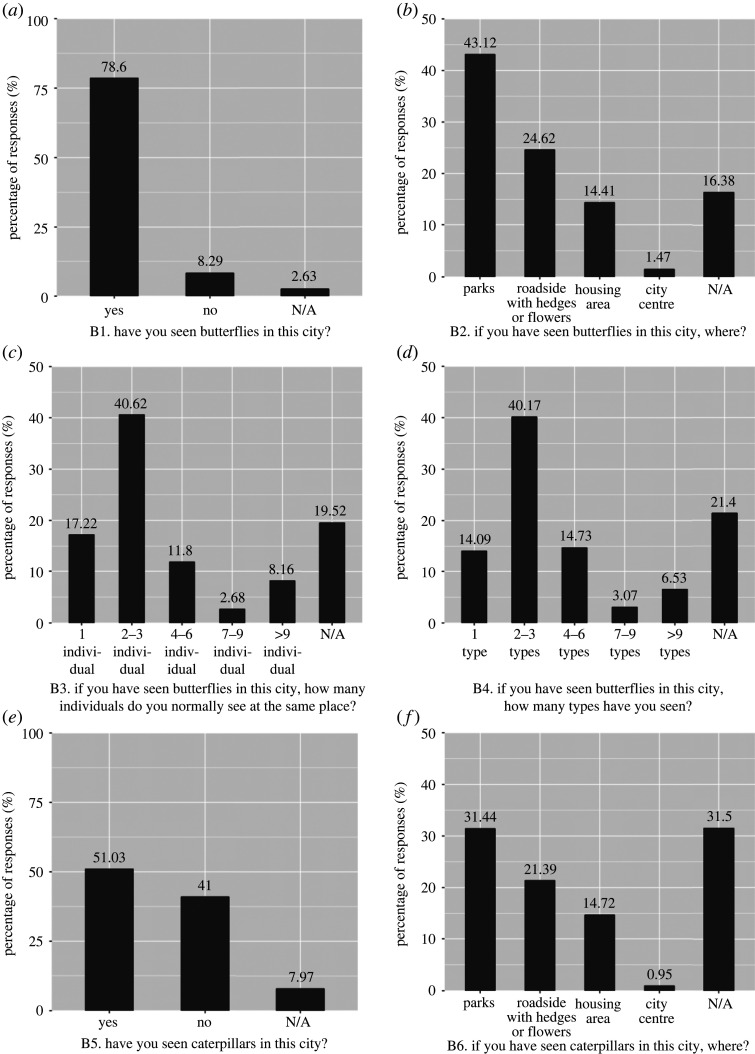
Responses to questions assessing familiarity with butterflies. For (*a*) and (*e*), the total responses were 1556. For (*b*), (*c*), (*d*) and (*f*), respondents could provide more than one answer; hence the responses were greater than 1556. N/A indicates ‘not answered’. Values on top of the bars represent the percentage of responses.

Overall, 48.78% of respondents visited natural places in their neighbourhood either ‘once a week’ or ‘almost daily’ (electronic supplementary material, table S1). The majority of the respondents (78.6%) reported having seen butterflies in the city. Of these respondents, the majority had seen butterflies at parks, mostly two to three individual butterflies at once, and two to three different types of butterflies at the same place (figure 3). Half of the respondents (51.03%) answered that they had seen caterpillars in the city. Of these respondents, most had seen caterpillars at parks (31.44%, figure 3).

**Figure 4.**
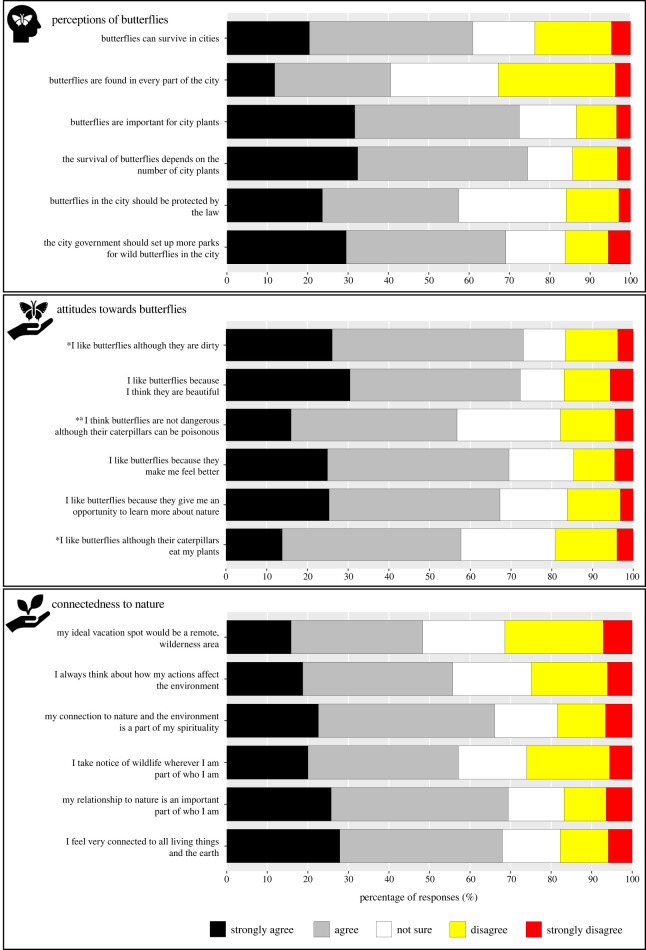
Perception of butterflies, attitude toward butterflies and connectedness to nature. *These statements were originally negative but were reversely scored here and for the linear mixed effects modelling. ^a^This statement was removed from statistical analyses due to low Cronbach's alpha value.

The dominance of ‘strongly agree’ and ‘agree’ responses to the statements in Parts C, D and E demonstrated generally positive perceptions of butterflies, attitudes toward butterflies and connectedness to nature among the respondents (figure 4).

**Figure 5.**
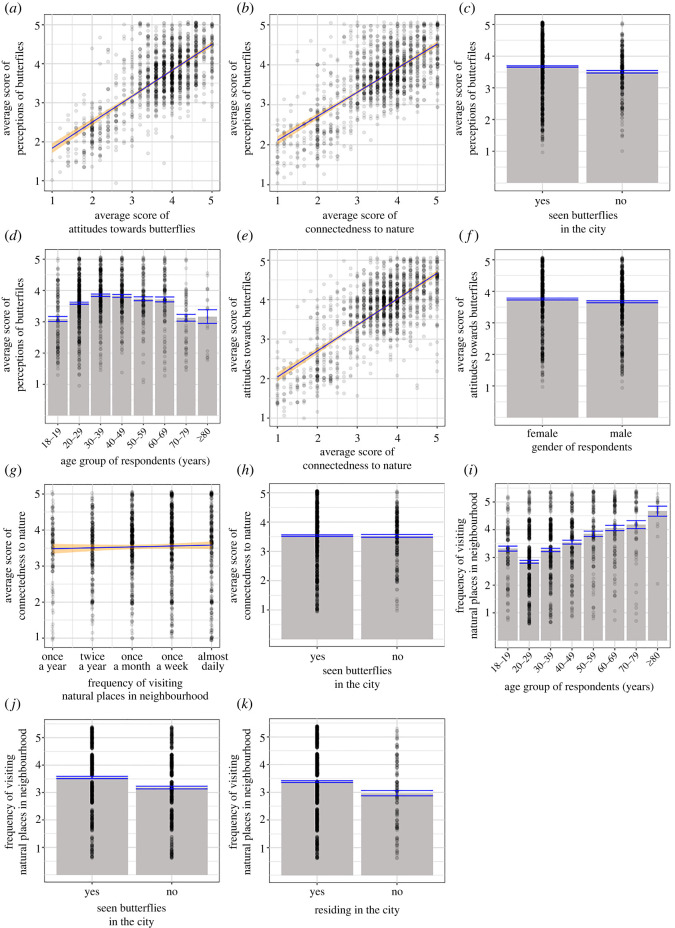
Visualization of responses based on the four linear mixed-effect models. For Model 1, perception of butterflies was plotted against (*a*) attitudes toward butterflies, (*b*) connectedness to nature, (*c*) sighting butterflies in the city and (*d*) age group of respondents. For Model 2, attitudes toward butterflies was plotted against (*a*) perceptions of butterflies, (*e*) connectedness to nature and (*f*) gender of respondents. For Model 3, connectedness to nature was plotted against (*g*) frequency of visiting natural places in neighbourhood and (*h*) sighting of butterflies in the city. For Model 4, frequency of visiting natural places in neighbourhood was plotted against (*i*) age group of respondents, (*j*) sighting of caterpillars in the city and (*k*) being resident of the city. Line of best fit (blue) with 99% confidence interval (orange) was plotted for (*a*), (*b*), (*e*) and (*g*). Responses were superimposed on predicted group mean (grey column) and standard error of means (blue bar) for (*c*), (*d*), (*f*), (*h*), (*i*), (*j*) and (*k*). ‘N/A’ responses were excluded from these plots.

**Figure 6.**
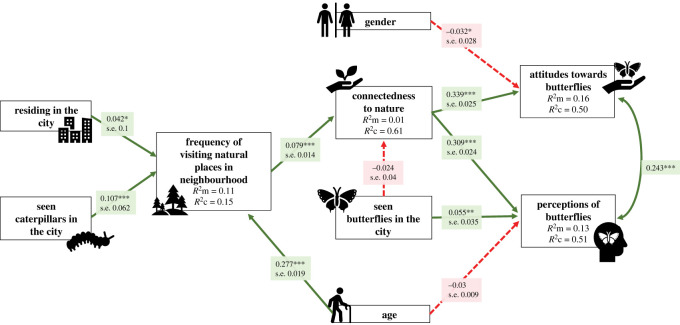
Piecewise structural equation modelling showing the standardized pathways for all response and explanatory variables based on coefficient estimates. The arrowhead indicates the pathway of the relationship, where one variable influenced the other. Green arrows with solid lines indicate a positive relationship between the two variables whereas red arrows with dashed lines indicate a negative relationship between the two variables. Note that the variables with binary responses were coded as: male/yes = 1; female/no = 0. Asterisks represent the significance levels of *p*-values in increasing order: *p < 0.05, **p < 0.01 and ***p < 0.001. *R*^2^*m* indicates marginal *R*^2^, *R*^2^c indicates conditional *R*^2^ whereas s.e. indicates standard error. Note that *p*-values > 0.05 indicated that the variables in the model were conditionally independent and all potential significant paths were included in the model.

Based on four models that resulted from LMM analysis (electronic supplementary material, table S2, table S3; and figure 5), several SEM models were constructed and compared. The SEM model with lowest AIC value (AIC = 65.223), a *p*-value > 0.05 for all directed separation tests, a *p*-value > 0.05 for Fisher's C test (21.223, *p* = 0.881, d.f. = 30) and that fitted the data well in *post hoc* assessments (electronic supplementary material, table S4) was retained and visualized (figure 6).

The SEM model (figure 6) confirmed that ‘perceptions of butterflies' and ‘attitudes toward butterflies’ had a significant positive association (*p* < 0.001; figure 5*a*). ‘Perception of butterflies' was positively associated with ‘connectedness to nature’ (*p* < 0.001; figure 5*b*) and ‘seen butterflies in the city’ (*p* = 0.001; figure 5*c*), but negatively, yet not significantly, associated with ‘age’ (*p* = 0.085; figure 5*d*). ‘Attitudes toward butterflies' was positively associated with ‘connectedness to nature’ (*p* < 0.001; figure 5*e*) and negatively associated, albeit weakly, with ‘gender’ (*p* = 0.049; figure 5*f*). ‘Connectedness to nature’ was positively associated with ‘frequency of visiting natural places in neighbourhood’ (*p* < 0.001; figure 5g), but negatively, yet not significantly, associated with ‘seen butterflies in the city’ (*p* = 0.147; figure 5*h*). ‘Frequency of visiting natural places in the neighbourhood’ was positively associated with ‘age’ (*p* < 0.001; figure 5*i*), ‘seen caterpillars in the city’ (*p* < 0.000; figure 5*j*) and ‘residing in the city’ (*p* = 0.013; figure 5*k*).

**Figure 7.**
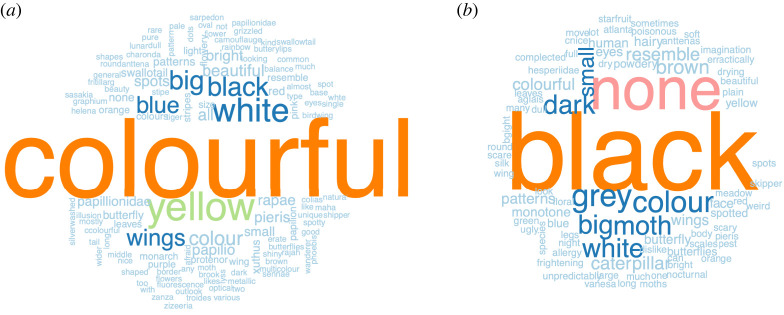
Qualitative responses to questions where respondents were asked to describe their preferred (*a*) and disliked (*b*) characteristics of butterflies.

We received 588 responses to the question about preferred characteristics of butterflies. The top ten keywords for the preferred characteristics of butterflies were ‘colourful’ (frequency = 211), ‘yellow’ (frequency = 67), ‘white’ (frequency = 51), ‘black’ (frequency = 39), ‘big’ (frequency = 38), ‘blue’ (frequency = 33), ‘wings’ (frequency = 32), ‘colour’ (frequency = 26), ‘beautiful’ (frequency = 18), and ‘all’ (frequency = 17) (figure 7*a*). For the question about disliked characteristics of butterflies, 300 responses were received. The top 10 keywords for the disliked characteristics of butterflies were ‘black’ (frequency = 85), ‘none’ (frequency = 43), ‘grey’ (frequency = 20), ‘colour’ (frequency = 18), ‘big’ (frequency = 16), ‘moth’ (frequency = 16), ‘dark’ (frequency = 15), ‘white’ (frequency = 15), ‘small’ (frequency = 11) and ‘brown’ (frequency = 10) (figure 7*b*).

## 4. Discussion

Perceptions of, and attitudes toward, wildlife are thought to be affected by exposure to, and direct experiences with, nature [8,12] which shape public support for wildlife conservation through the ‘nature benefit’ hypothesis [20]. However, no large-scale assessment of public familiarity with, perceptions of, or attitudes toward nature has been conducted across East and Southeast Asia's megacities, where highly urbanized environments are often considered to offer little opportunity for human–nature interactions. Here, we provide empirical evidence of how human–wildlife interactions can shape increased perceptions of, and positive attitudes toward, nature even in the most urbanized places on the planet. In these megacities, butterflies are ideal for representing ‘urban wildlife’ because previous studies have recorded high species richness and abundance of butterflies in their urban parks [26–31,49–51] including common and conspicuous pierids (*Appias* spp., *Catopsilia* spp*.*, *Pieris* spp.).

As butterflies are ubiquitous, it was reassuring that 79% of respondents told us they had seen butterflies, indicating widespread familiarity with butterflies, with most sightings taking place in parks in their cities. As the questionnaires were conducted solely in urban parks (including botanical gardens), we would not have reached those who never visit natural places in their neighbourhood. However, surveys in Hong Kong found that only 8% of respondents had never visited parks [52], and the length of walking distance to a park is the major factor determining their accessibility [53]. Another study which overlapped with four of these megacities reported 55% of their respondents have seen bees in the city [33]. This is lower than the percentage that had seen butterflies (78%) but is similar to the percentage who had seen caterpillars (51%) in our survey. This may be expected as seeing bees or caterpillars would usually require getting closer to vegetation, whereas butterflies can be observed from distances and flying across open areas. Our survey also found that the frequency of visiting natural places in the neighbourhood was positively associated with sightings of caterpillars suggesting that the more regularly park users visit natural places, the more likely they become familiar with the wildlife they support, including the inconspicuous elements such as caterpillars.

**Figure 8.**
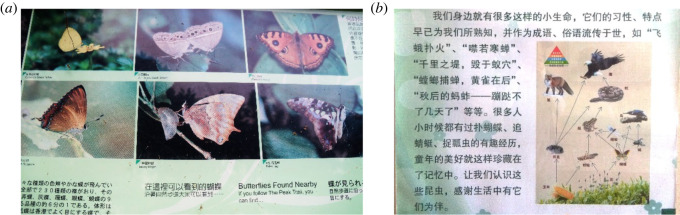
(*a*) Part of an information board at Kadoorie Farm and Botanical Garden in Hong Kong, SAR, showing butterflies found nearby and (*b*) part of an information board from Zhongshan Park, Beijing (both photographs taken by J.-J.W.).

The City Nature Challenge is an international event tasked with motivating people around the world to document wildlife in their cities and submit their observations through an online platform (www.inaturalist.org). The competition has seen high participation levels in East and Southeast Asia in 2022 (https://citynaturechallenge.org/collective-results-2022/). During the international challenge in 2021, butterflies were frequently recorded in Greater Kuala Lumpur (Klang Valley), including the common species (i.e. *Acraea terpsicore*, *Junonia almana*, *Zizina otis*; https://www.inaturalist.org/projects/city-nature-challenge-2021-klang-valley). These common butterfly species are easy to distinguish with little knowledge required to identify them taxonomically. In our study, 78% of respondents who had seen butterflies could distinguish multiple types of butterflies, suggesting some species recognition capacity among the general public (see [31,54]), who are thought to generally underestimate species richness [55]. Such species recognition capacity among the public can be further enhanced by setting up information boards within parks (figure 8*a*), which has already been used to promote public familiarity with butterflies in urban parks across East and Southeast Asia.

In our survey, the locations where respondents were most likely to report having seen butterflies in the city were urban parks. This is not surprising considering that we used ‘park’ broadly in our questionnaire to include botanical and zoological gardens, and university campuses, as these are the major green spaces in cities. Similar to birds [56], it is likely that the majority of human–butterfly interactions in cities take place in urban parks. As urban residents in East and Southeast Asia generally live in apartments and do not have access to private gardens, urban parks are heavily used for exercising opportunities [57], to ‘get fresh air’ [58] and for ‘picnics’ [59]. Such leisure activities can provide a pathway to familiarity with, and connectedness to nature [60]. Park characteristics (e.g. size, age, distance to wild areas, presence of flowering plants) will affect the abundance and diversity of butterflies in each park [28,29,51], and consequently, park users' familiarity with the butterflies. Therefore, park management strategies such as maintaining a large proportion of native or original vegetation as ‘unmanaged’ or ‘wilderness’ space as part of ‘ecological parks’ in cities [28,29,51,61] can be used to promote the presence of butterflies and other wildlife in the cities. Our respondents also reported sightings of butterflies and caterpillars along roadsides with hedges or flowers. The presence of butterflies and other wildlife along roadsides could also be enhanced by the ‘garden city’ concept already implemented in a few cities in East and Southeast Asia [51,62].

High levels of familiarity with butterflies can result in greater perceptions of butterflies, as revealed by our survey. The statements in our questionnaire were phrased in the manner to relate perceptions of butterflies to recognition of butterflies as an integral part of the city. Perception of butterflies was generally high among respondents, and in particular, the relationship between butterflies and plants seemed to be well understood. Many respondents had seen butterflies not only in parks but along roadsides and in housing areas, and those who have high level of connectedness to nature exhibited positive perceptions of butterflies, indicating a public recognition that butterflies are part of the ‘nature’ in the city. Other studies have highlighted that ‘nature’ has often been portrayed to children as separate from ‘culture’, sitting outside of human influence [17], resulting in urban residents lacking a comprehensive understanding of wildlife and any positive views held being primarily abstract [63]. Increased perception of wildlife can be generated through increased knowledge and awareness, and other researchers have proposed outreach and education programmes targeted at specific demographics [17,25]. Educational materials placed in urban parks showing butterflies as integral parts of city ecosystems have already been deployed in some megacities as an awareness and education strategy targeting park users (figure 8*b*).

Despite positive attitudes (i.e. expressions of liking) toward nature being widely held and expressed globally, not every component of biodiversity will be equally popular, or perceived as ecologically meaningful to a city [64]. This may especially be the case in tropical regions. Urban wildlife such as pythons, macaques [65], bats [25], birds [17] and insects generally [33,64] are often regarded as dangerous or nuisances. Unlike some urban wildlife, butterflies cannot bite and are not known to transmit any human diseases, and therefore, butterflies are not generally subject to human–wildlife conflicts in urban areas. However, maintaining the ‘unmanaged’ or ‘wilderness’ areas in urban parks to promote butterfly diversity [28,29,51,61] may be viewed negatively by some urban residents due to the close proximity of the ‘wilderness’ to their neighbourhood [64].

In our survey, more than 50% of respondents expressed a liking for butterflies despite a general understanding of ecosystem disservices associated with butterflies and caterpillars (i.e. plant damage and potential health risks). Studies have suggested that East Asian attitudes toward animals are converging with their Western counterparts [66], with a greater aesthetic appreciation of animals (e.g. [67]). This is supported by the results from our survey where 70% of the respondents reported that they liked butterflies because they were beautiful, and mentioned a range of colour characteristics and ‘spots’ when describing their preferred traits of butterflies. A study from the United States found that measures of liking butterflies were significantly higher for species with eyespots [68]. This suggests butterflies may be particularly suited as ambassadors of urban wildlife through exploiting their aesthetic appeal, with a particular focus on species with eyespots (e.g. Nymphalinae and Satyrinae) and a potential role for visual media (e.g. coffee table books) in conservation initiatives [35,69].

In East and Southeast Asia, urban parks are probably the most commonly visited outdoor spaces [70]. Our survey found that the frequency of visiting urban natural places was positively associated with connectedness to nature. Although residents may not visit parks with the expressed purpose of connecting with nature, visiting parks for leisure activities provides a unique opportunity for urban residents to experience and familiarize themselves with wildlife, passively developing a connectedness to nature [60]. Feeling connected to nature through simple everyday engagement plays a critical role in pro-environmental behaviour [16,71], implicating urban parks as essential for developing this connection. However, there is evidence that actively pursuing and engaging in nature-based activities (e.g. hiking, viewing wildlife, gardening) is pivotal in developing deeper levels of connectedness to nature [72]. Lack of access to parks is therefore a barrier to connectedness with nature which needs to be addressed through urban planning. But, there is also a need for outreach events to facilitate and encourage park visits, particularly targeting those who are less likely to visit green spaces and engage with nature-related activities such as younger and older people, those with lower incomes, and without university education who are less likely to visit green spaces and engage with nature-related activities [70]. It is also imperative to encourage visitors with young children, as research has shown that early exposure to nature develops positive environmental attitudes in children and builds a sense of nature connectedness in later life [12,65,72].

Across our analyses, the strongest relationship we found was that between attitudes toward butterflies and connectedness to nature, followed by the relationship between perceptions of butterflies and connectedness to nature. This finding firmly links parks users' thoughts and feelings about butterflies with a general world view of nature. Therefore, this suggests butterflies in urban parks can play a key role in building connectedness to nature and in turn pro-environmental behaviours, which requires action in three main areas:

(1) *Enhancing butterfly diversity in parks.* Parks should be managed and maintained in ways that promote butterfly diversity, and enabling parks users to experience and familiarize themselves with butterflies. This may be through the provision of ‘unmanaged’ or ‘wilderness’ patches in parks [28,29,51] and through the planting of native and non-native plants as food and breeding sites for butterflies [73].
(2) *Raising public awareness.* Educational materials such as information boards and pocket guides should be available at parks to encourage familiarity with butterflies and to improve perceptions of butterflies. This could extend to butterfly houses, butterfly rearing facilities and even small museums in parks (e.g. Queen Sirikit Botanic Garden, Bangkok).
(3) *Involving the public.* Butterflies should be ‘exploited’ as ideal umbrella species when conducting outreach and educational activities that intentionally connect urban residents with nature in the cities, such as citizen science and habitat creation volunteering projects [74]. This will result in pro-environmental behaviours [74].

## Data Availability

The data are provided in electronic supplementary material [75].

## References

[1] Wigginton NS, Fahrenkamp-Uppenbrink J, Wible B, Malakoff D. 2016 Cities are the future. Science **352**, 904-905. (10.1126/science.352.6288.904) 27199407

[2] Schneider A et al. 2015 A new urban landscape in East–Southeast Asia, 2000–2010. Environ. Res. Lett. **10**, 034 002. (10.1088/1748-9326/10/3/034002)

[3] Yeh AG, Chen Z. 2020 From cities to super mega city regions in China in a new wave of urbanisation and economic transition: issues and challenges. Urban Stud. **57**, 636-654. (10.1177/0042098019879566)

[4] McKinney ML. 2002 Urbanization, biodiversity, and conservation: the impacts of urbanization on native species are poorly studied, but educating a highly urbanized human population about these impacts can greatly improve species conservation in all ecosystems. BioScience **52**, 883-890. (10.1641/0006-3568(2002)052[0883:UBAC]2.0.CO;2)

[5] Lundholm JT, Richardson PJ. 2010 Habitat analogues for reconciliation ecology in urban and industrial environments. J. Appl. Ecol. **47**, 966-975. (10.1111/j.1365-2664.2010.01857.x)

[6] Keeler BL et al. 2019 Social-ecological and technological factors moderate the value of urban nature. Nat. Sustain. **2**, 29-38. (10.1038/s41893-018-0202-1)

[7] Lepczyk CA, Aronson MF, Evans KL, Goddard MA, Lerman SB, MacIvor JS. 2017 Biodiversity in the city: fundamental questions for understanding the ecology of urban green spaces for biodiversity conservation. BioScience **67**, 799-807. (10.1093/biosci/bix079)

[8] Dunn RR, Gavin MC, Sanchez MC, Solomon JN. 2006 The pigeon paradox: dependence of global conservation on urban nature. Conserv. Biol. **20**, 1814-1816. (10.1111/j.1523-1739.2006.00533.x) 17181818

[9] Belaire JA, Westphal LM, Whelan CJ, Minor ES. 2015 Urban residents' perceptions of birds in the neighborhood: biodiversity, cultural ecosystem services, and disservices. Condor **117**, 192-202. (10.1650/CONDOR-14-128.1)

[10] Campbell-Arvai V. 2019 Engaging urban nature: improving our understanding of public perceptions of the role of biodiversity in cities. Urban Ecosyst. **22**, 409-423. (10.1007/s11252-018-0821-3)

[11] Fukano Y, Soga M. 2021 Why do so many modern people hate insects? The urbanization–disgust hypothesis. Sci. Total Environ. **777**, 146229. (10.1016/j.scitotenv.2021.146229)

[12] Soga M, Gaston KJ, Koyanagi TF, Kurisu K, Hanaki K. 2016 Urban residents’ perceptions of neighbourhood nature: does the extinction of experience matter? Biol. Conserv. **203**, 143-150. (10.1016/j.biocon.2016.09.020)

[13] Miller JR. 2006 Restoration, reconciliation, and reconnecting with nature nearby. Biol. Conserv. **127**, 356-361. (10.1016/j.biocon.2005.07.021)

[14] Soga M, Gaston KJ. 2016 Extinction of experience: the loss of human–nature interactions. Front. Ecol. Environ. **14**, 94-101. (10.1002/fee.1225)

[15] Cameron RW et al. 2020 Where the wild things are! Do urban green spaces with greater avian biodiversity promote more positive emotions in humans? Urban Ecosyst. **23**, 301-317. (10.1007/s11252-020-00929-z)

[16] Alcock I, White MP, Pahl S, Duarte-Davidson R, Fleming LE. 2020 Associations between pro-environmental behaviour and neighbourhood nature, nature visit frequency and nature appreciation: evidence from a nationally representative survey in England. Environ. Int. **136**, 105441. (10.1016/j.envint.2019.105441) 31927464

[17] Leong RA, Fung TK, Sachidhanandam U, Drillet Z, Edwards PJ, Richards DR. 2020 Use of structural equation modeling to explore influences on perceptions of ecosystem services and disservices attributed to birds in Singapore. Ecosyst. Serv. **46**, 101211. (10.1016/j.ecoser.2020.101211)

[18] Kingston T. 2016 Cute, creepy, or crispy—how values, attitudes, and norms shape human behavior toward bats. In Bats in the anthropocene: conservation of bats in a changing world (eds CC Voigt, T Kingston), pp. 571-595. Cham, Switzerland: Springer.

[19] Hwang YH, Jain A. 2021 Landscape design approaches to enhance human–wildlife interactions in a compact tropical city. J. Urban Ecol. **7**, juab007. (10.1093/jue/juab007)

[20] Soga M, Gaston KJ. 2021 Towards a unified understanding of human–nature interactions. Nat. Sustain. **5**, 374-383. (10.1038/s41893-021-00818-z)

[21] Forth G. 2017 What a little bird tells us about symbolic thought: the russet-capped stubtail (*Tesia everetti*) in Nage augury, myth, and metaphor. J. Ethnobiol. **37**, 682-699. (10.2993/0278-0771-37.4.682)

[22] Perry G, Boal C, Verble R, Wallace M. 2020 ‘Good’ and ‘bad’ urban wildlife. In Problematic wildlife II (eds FM Angelici, L Rossi), pp. 141-170. Cham, Switzerland: Springer.

[23] Low MR et al. 2021 Bane or blessing? Reviewing cultural values of bats across the Asia-Pacific Region. J Ethnobiol. **41**, 18-34. (10.2993/0278-0771-41.1.18)

[24] Areaya H, Haileselasie TH. 2013 Knowledge and attitude of peasants towards birds in church forests in Tigray region, Northern Ethiopia. Int. J. Biodivers. Conserv. **5**, 461-468. (10.5897/IJBC2013.0554)

[25] Lim VC, Wilson JJ. 2019 Public perceptions and knowledge of, and responses to, bats in urban areas in peninsular Malaysia. Anthrozoös **32**, 825-834. (10.1080/08927936.2019.1673063)

[26] Jain A, Chan SK, Vlasanek P, Webb EL. 2020 Impacts of habitat on butterfly dispersal in tropical forests, parks and grassland patches embedded in an urban landscape. Biotropica **52**, 404-409. (10.1111/btp.12760)

[27] Jaturas N, Sing KW, Wilson JJ, Dong H. 2020 Butterflies in urban parks in the Bangkok Metropolitan Region, Thailand. Biodivers. Data J. **8**, e56317. (10.3897/BDJ.8.e56317) 33117077 PMC7572522

[28] Sing KW, Jusoh WF, Hashim NR, Wilson JJ. 2016 Urban parks: refuges for tropical butterflies in Southeast Asia? Urban Ecosyst. **19**, 1131-1147. (10.1007/s11252-016-0542-4)

[29] Sing KW, Luo J, Wang W, Jaturas N, Soga M, Yang X, Dong H, Wilson JJ. 2019 Ring roads and urban biodiversity: distribution of butterflies in urban parks in Beijing city and correlations with other indicator species. Sci. Rep. **9**, 7653. (10.1038/s41598-019-43997-8) 31113976 PMC6529450

[30] Tsang TP, Bonebrake TC. 2017 Contrasting roles of environmental and spatial processes for common and rare urban butterfly species compositions. Landsc. Ecol. **32**, 47-57. (10.1007/s10980-016-0427-1)

[31] Wilson JJ, Jisming-See SW, Brandon-Mong GJ, Lim AH, Lim VC, Lee PS, Sing KW. 2015 Citizen science: the first Peninsular Malaysia butterfly count. Biodivers. Data J. **3**, e7159. (10.3897/BDJ.3.e7159) PMC4700385 26751033

[32] Ryan SF et al. 2019 Global invasion history of the agricultural pest butterfly *Pieris rapae* revealed with genomics and citizen science. Proc. Natl Acad. Sci. USA **116**, 20 015-20 024. (10.1073/pnas.1907492116) 31506352 PMC6778179

[33] Sing KW, Wang WZ, Wan T, Lee PS, Li ZX, Chen X, Wang YY, Wilson JJ. 2016 Diversity and human perceptions of bees (Hymenoptera: Apoidea) in Southeast Asian megacities. Genome **59**, 827-839. (10.1139/gen-2015-0159) 27327818

[34] Oh RR, Fielding KS, Carrasco RL, Fuller RA. 2020 No evidence of an extinction of experience or emotional disconnect from nature in urban Singapore. People Nat. **2**, 1196-1209. (10.1002/pan3.10148)

[35] Lim VC, Justine EV, Yusof K, Wan Mohamad Ariffin WN, Goh HC, Fadzil KS. 2021 Eliciting local knowledge of ecosystem services using participatory mapping and Photovoice: a case study of Tun Mustapha Park, Malaysia. PLoS ONE **16**, e0253740. (10.1371/journal.pone.0253740) 34242233 PMC8270451

[36] Nisbet EK, Zelenski JM. 2013 The NR-6: a new brief measure of nature relatedness. Front. Psychol. **4**, 813. (10.3389/fpsyg.2013.00813) 24198806 PMC3814587

[37] R Core Team. 2020 R: a language and environment for statistical computing. Vienna, Austria: R Foundation for Statistical Computing. See http://www.R-project.org/.

[38] Fellows I. 2018 wordcloud. R package version 2.6. The Comprehensive R Archive Network. See https://cran.r-project.org/web/packages/wordcloud/index.html.

[39] Rizopoulos D. 2006 ltm: an R package for latent variable modeling and item response analysis. J. Stat. Softw. **17**, 1-25. (10.18637/jss.v017.i05)

[40] Tavakol M, Dennick R. 2011 Making sense of Cronbach's alpha. Int. J. Med. Educ. **2**, 53-55. (10.5116/ijme.4dfb.8dfd) 28029643 PMC4205511

[41] Bates D, Mächler M, Bolker B, Walker S. 2014 lme4: linear mixed-effects models using Eigen and S4. R package version 1.1–7. See http://CRAN.R-project.org/package=lme4.

[42] Bartoń K. 2020 MuMIn: multi-model inference. R package version 1.43.17. See https://CRAN.R-project.org/package=MuMIn.

[43] Frank J, Fabozzi FJ, Focardi SM, Rachev ST, Arshanapalli BG. 2014 The basics of financial econometrics: tools, concepts, and asset management applications. Hoboken, NJ: John Wiley & Sons.

[44] Lefcheck JS. 2016 piecewiseSEM: piecewise structural equation modelling in R for ecology, evolution, and systematics. Methods Ecol. Evol. **7**, 573-579. (10.1111/2041-210X.12512)

[45] Lefcheck JS. 2021 3.4 Model fitting using piecewiseSEM. GitHub. See https://jslefche.github.io/sem_book/local-estimation.html#model-fitting-using-piecewisesem.

[46] Rosseel Y et al. 2021 lavaan: latent variable analysis. R package version 0.6-8. See https://CRAN.R-project.org/package=lavaan.

[47] Shi D, Lee T, Maydeu-Olivares A. 2019 Understanding the model size effect on SEM fit indices. Educ. Psychol. Meas. **79**, 310-334. (10.1177/0013164418783530) 30911195 PMC6425088

[48] Cho G, Hwang H, Sarstedt M, Ringle CM. 2020 Cutoff criteria for overall model fit indexes in generalized structured component analysis. J. Mark. Anal. **8**, 189-202. (10.1057/s41270-020-00089-1)

[49] Soga M, Yamaura Y, Koike S, Gaston KJ. 2014 Land sharing vs. land sparing: does the compact city reconcile urban development and biodiversity conservation? J. Appl. Ecol. **51**, 1378-1386. (10.1111/1365-2664.12280)

[50] Soga M, Yamaura Y, Koike S, Gaston KJ. 2014 Woodland remnants as an urban wildlife refuge: a cross-taxonomic assessment. Biodivers. Conserv. **23**, 649-659. (10.1007/s10531-014-0622-9)

[51] Sing KW, Dong H, Wang WZ, Wilson JJ. 2016 Can butterflies cope with city life? Butterfly diversity in a young megacity in southern China. Genome **59**, 751-761. (10.1139/gen-2015-0192) 27314400

[52] Wong KK. 2009 Urban park visiting habits and leisure activities of residents in Hong Kong, China. Manag. Leis. **14**, 125-140. (10.1080/13606710902752653)

[53] Tian Y, Jim CY, Liu Y. 2017 Using a spatial interaction model to assess the accessibility of district parks in Hong Kong. Sustainability **9**, 1924. (10.3390/su9111924)

[54] Jisming-See SW, Sing KW, Wilson JJ. 2016 DNA barcodes and citizen science provoke a diversity reappraisal for the ‘ring’ butterflies of Peninsular Malaysia (Ypthima: Satyrinae: Nymphalidae: Lepidoptera). Genome **59**, 879-888. (10.1139/gen-2015-0156) 27333330

[55] Shwartz A, Turbé A, Simon L, Julliard R. 2014 Enhancing urban biodiversity and its influence on city-dwellers: an experiment. Biol. Conserv. **171**, 82-90. (10.1016/j.biocon.2014.01.009)

[56] Zhang Z, Huang G. 2020 How do urban parks provide bird habitats and birdwatching service? Evidence from Beijing, China. Remote Sens. **12**, 3166. (10.3390/rs12193166)

[57] Chow BC, McKenzie TL, Sit CH. 2016 Public parks in Hong Kong: characteristics of physical activity areas and their users. Int. J. Environ. Res. Public Health **13**, 639. (10.3390/ijerph13070639) 27367709 PMC4962180

[58] Sreetheran M. 2017 Exploring the urban park use, preference and behaviours among the residents of Kuala Lumpur, Malaysia. Urban For. Urban Green. **25**, 85-93. (10.1016/j.ufug.2017.05.003)

[59] Schetke S, Qureshi S, Lautenbach S, Kabisch N. 2016 What determines the use of urban green spaces in highly urbanized areas? Examples from two fast growing Asian cities. Urban For. Urban Green. **16**, 150-159. (10.1016/j.ufug.2016.02.009)

[60] Mateer TJ. 2022 Developing connectedness to nature in urban outdoor settings: a potential pathway through awe, solitude, and leisure. Front. Psychol. **13**, 4137. (10.3389/fpsyg.2022.940939) PMC9309726 35898979

[61] Wang WL, Suman DO, Zhang HH, Xu ZB, Ma FZ, Hu SJ. 2020 Butterfly conservation in China: from science to action. Insects **11**, 661. (10.3390/insects11100661) 32992975 PMC7600441

[62] Kuo L. 2019 Chengdu is blossoming as China's ‘park city’, but its residents pay the price of beautification. *South China Morning Post*. See https://www.scmp.com/magazines/post-magazine/long-reads/article/2185246/chengdu-blossoming-chinas-park-city.

[63] Kato E, Yano Y, Ohe Y. 2019 Investigating gaps in perception of wildlife between urban and rural inhabitants: empirical evidence from Japan. Sustainability **11**, 4516. (10.3390/su11174516)

[64] Kajzer-Bonk J, Nowicki P. 2022 Butterflies in trouble: the effectiveness of Natura 2000 network in preventing habitat loss and population declines of endangered species in urban area. Ecol. Indic. **135**, 108518. (10.1016/j.ecolind.2021.108518)

[65] Ngo KM, Hosaka T, Numata S. 2019 The influence of childhood nature experience on attitudes and tolerance towards problem-causing animals in Singapore. Urban For. Urban Green. **41**, 150-157. (10.1016/j.ufug.2019.04.003)

[66] Gao J, Zhang C, Huang Z. 2018 Chinese tourists' views of nature and natural landscape interpretation: a generational perspective. J. Sustain. Tour. **26**, 668-684. (10.1080/09669582.2017.1377722)

[67] Basak SM, Hossain MS, O'Mahony DT, Okarma H, Widera E, Wierzbowska IA. 2022 Public perceptions and attitudes toward urban wildlife encounters—a decade of change. Sci. Total Environ. **834**, 155603. (10.1016/j.scitotenv.2022.155603) 35523348

[68] Manesi Z, Van Lange PA, Pollet TV. 2015 Butterfly eyespots: their potential influence on aesthetic preferences and conservation attitudes. PLoS ONE **10**, e0141433. (10.1371/journal.pone.0141433) 26544692 PMC4636354

[69] Farnsworth BE. 2011 Conservation photography as environmental education: focus on the pedagogues. Environ. Educ. Res. **17**, 769-787. (10.1080/13504622.2011.618627)

[70] Richards DR, Fung TK, Leong RA, Sachidhanandam U, Drillet Z, Edwards PJ. 2020 Demographic biases in engagement with nature in a tropical Asian city. PLoS ONE **15**, e0231576. (10.1371/journal.pone.0231576) 32339175 PMC7185705

[71] Richardson M, Passmore HA, Barbett L, Lumber R, Thomas R, Hunt A. 2020 The green care code: how nature connectedness and simple activities help explain pro-nature conservation behaviours. People Nat. **2**, 821-839. (10.1002/pan3.10117)

[72] Wright PA, Matthews C. 2015 Building a culture of conservation: research findings and research priorities on connecting people to nature in parks. Parks **21**, 11-24. (10.2305/IUCN.CH.2014.PARKS-21-2PAW.en)

[73] Jain A, Zeng Y, Webb EL. 2021 Critical dependence of butterflies on a non-native host plant in the urban tropics. Front. Ecol. Evol. **9**, 655012. (10.3389/fevo.2021.655012)

[74] Lewandowski EJ, Oberhauser KS. 2017 Contributions of citizen scientists and habitat volunteers to monarch butterfly conservation. Hum. Dimen. Wildl. **22**, 55-70. (10.1080/10871209.2017.1250293)

[75] Lim V-C et al. 2022 Data from: Familiarity with, perceptions of and attitudes toward butterflies of urban park users in megacities across East and Southeast Asia. *Figshare*. (10.6084/m9.figshare.c.6266188) PMC9653264 36405642

